# Elevated CXCL1 increases hepatocellular carcinoma aggressiveness and is inhibited by miRNA-200a

**DOI:** 10.18632/oncotarget.11350

**Published:** 2016-08-17

**Authors:** Xiao Cui, Zhao Li, Jie Gao, Peng-Ji Gao, Yan-bing Ni, Ji-Ye Zhu

**Affiliations:** ^1^ Department of Hepatobilliary Surgery, Beijing Key Surgical Basic Research Laboratory of Liver Cirrhosis and Liver Cancer, Peking University People's Hospital, Beijing 100044, China; ^2^ Department of General Surgery, The Second Hospital of Anhui Medical University, Hefei 230601, China

**Keywords:** CXCL1, carcinogenesis, hepatocellular carcinoma, miR-200a

## Abstract

In this study, we investigated the value of measurement of the chemokine CXCL1 in clinical management of hepatocellular carcinoma (HCC) and its possible role in the molecular pathogenesis of HCC. High CXCL1 expression predicted recurrence in HCC patients and promoted tumor progression in both *in vivo* and *in vitro* experimental systems. Overexpression of CXCL1 increased mitochondrial metabolism and activated the epithelial-to-mesenchymal transition (EMT). Using computational analysis we identified the microRNA miR-200a as a putative post-transcriptional regulator of CXCL1. We found that levels of miR-200a were inversely correlated with CXCL1 expression in HCC patient tissue samples by northern blot and qRT-PCR. Furthermore, CXCL1 was identified as a direct target which was bound and inhibited by miR- 200a. These findings provide new insights into the role of CXCL1 in HCC and its post-transcriptional regulation and suggest it may be a prognostic indicator for poor outcomes and a potential target for therapy.

## INTRODUCTION

Hepatocellular carcinoma (HCC) ranks as the third leading cause of cancer-related mortalities. Globally, nearly 750,000 new patients are diagnosed with liver cancer each year [1]. Chronic liver inflammation caused by viral hepatitis is the primary risk factor for HCC. Although biologically-targeted therapies and surgical interventions have progressed, the overall 5 year recurrence rate after hepatectomy remains as high as 70% [2]. To provide new prognostic indicators and targeted therapies for improved clinical management, the underlying molecular mechanisms of HCC need to be further investigated.

Chemokines are small molecular weight proteins (8–13 KD) that drive the migration of a variety of immune cells [3]. CXCL1 (chemokine with C-X-C motif ligand 1) is a member of the CXC chemokine family and promotes neoplastic transformation, tumorigenesis, and angiogenesis in breast, lung, pancreatic, colorectal, bladder, and prostate cancer, as well as in melanoma, by binding specifically to CXCR2 [4–9]. Nonparenchymal liver cells, including hepatic stellate cells, hepatic dendritic cells, neutrophils, monocytes, and Kupffer cells, secrete CXCL1 and other chemokines to recruit immune cells and modify the HCC tumor microenvironment [10].

In this study, we investigated the association between CXCL1 expression and clinicopathologic parameters in HCC patients as well as the accuracy of CXCL1 levels in predicting prognosis after hepatectomy. We determined the effect of CXCL1 on HCC progression through lentiviral overexpression and knockdown in HCC cells. Additionally, we sought to identify regulators of CXCL1 activity in HCC by using computational analysis of publicly available data and subsequent lentiviral overexpression and knockdown.

## RESULTS

### Correlation between CXCL1 expression and HCC recurrence after hepatectomy

**Figure 1:**
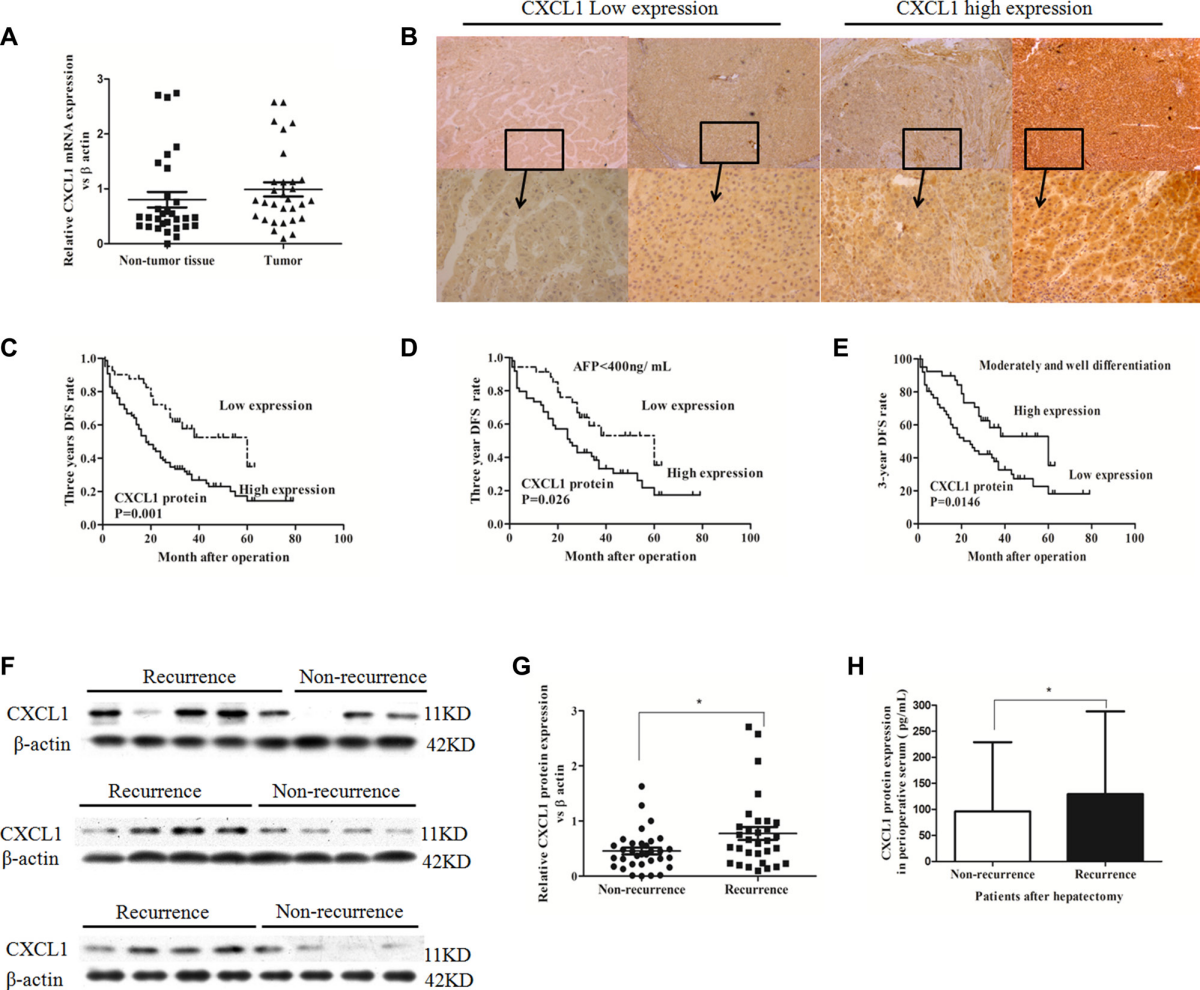
CXCL1 expression in HCC tumor and adjacent non-tumor tissues and association with recurrence (**A**) Comparison of mRNA expression of CXCL1 between thirty frozen tumor tissues and paired adjacent non-tumor tissues from HCC patients (*P* > 0.05). (**B**) Low and high CXCL1 expression staining scores in HCC. Original magnification was ×50 and ×200. (**C**) Patients with low CXCL1 expression in HCC tissues had higher 3 year DFS rates than those with high CXCL1 expression. (**D**) In serum AFP < 400 ng/mL subgroup, patients with low CXCL1 expression in HCC tissues had better 3 year DFS rates than patients with high CXCL1 expression. (**E**) In moderately and well–differentiated tumor subgroups, patients with low CXCL1 expression had better prognosis in 3 year DFS rates than patients with high expression. (**F**) CXCL1 protein expression in frozen HCC tissues. (**G**) The level of CXCL1 protein expression in patients with recurrence at 3 years after hepatectomy was higher than those without recurrence, which was confirmed by immunoblotting (, non-parameter test). (**H**) The level of CXCL1 protein in perioperative serum from patients with recurrence at 3 year was higher than those without recurrence, as assessed by ELISA. *P < 0.05, **P < 0.01. Results were presented as the average (mean ± SD) representing the average of three independent experiments.

A comparison analysis was performed using paired tissue samples from 30 patients, but no difference was observed in CXCL1 mRNA expression between tumor and adjacent non-tumor tissues (*P* = 0.945) (Figure 1A). In order to further investigate potential relationships, a microarray dataset assembled by Roessler et al. [11] was analyzed for CXCL1 expression in HCC patients with a history of hepatitis virus B (HBV) infection (236 / 247). In agreement with our findings, this analysis revealed no significant statistical differences in CXCL1 expression between tumor and adjacent non-tumor tissues (*P* = 0.441) (Supplementary Figure S1).

Immunoblotting, ELISA, and immunohistochemistry on clinical samples were performed and correlation with demographic data analyzed. In total, 119 HCC patients (age range: 33–83 years old) who had undergone hepatectomy at Peking University People's Hospital were enrolled. The median survival time was 35 months (total range: 1 to 89 months). The three-year survival rate was 66.4%, while the 3-year disease free survival (DFS) rate was 35.3%. The demographics of the patients are depicted in Table 1. No patients were lost in the follow-up period or died of diseases which were unassociated with HCC.

**Table 1:** Associations between CXCL1 expression and clinicopathologic variables of HCC patients n (%)

Clinical characteristic		CXCL1		*P*-value
	All cases (*n*= 119)	High expression	Low expression	
Gender(Female/Male)				0.712
Female	19	13 (16.9)	6 (14.3)	
Male	100	64 (83.1)	36 (85.7)	
Cirrhosis				0.156
No	18	9 (11.7)	9 (21.4)	
Yes	101	68 (88.3)	33 (78.6)	
AFP (≥ 400/< 400)				0.024
≥ 400 ng/mL	35	28 (36.4)	7 (16.7)	
< 400 ng/mL	84	49 (63.6)	35 (83.3)	
HBV-DNA (Positive/Negative)				0.982
Positive (≥ 5.0 × 10^2^)	65	35 (45.5)	19 (45.2)	
Negative (< 5.0 × 10^2^)	54	42 (54.5)	23 (54.8)	
Microvascular invasion (Yes/No)				0.321
Yes	32	23 (29.9)	9 (21.4)	
No	87	54 (70.1)	33 (78.6)	
TNM stage (I/II/III)				0.099
I	67	38 (49.4)	29 (69.0)	
II	28	20 (26.0)	8 (19.0)	
III	24	19 (24.7)	5 (11.9)	
Differentiation (Poorly/Moderately - Well)				0.001
Poorly	33	25 (32.5)	2 (4.8)	
Moderately - Well	86	52 (67.5)	40 (95.2)	
Tumor size (≥ 5 cm/< 5 cm)				0.683
≥ 5 cm	65	36 (46.8)	18 (42.9)	
< 5 cm	54	41 (53.2)	24 (57.1)	

Paraffin sections of samples from the 119 patients were analyzed by immunohistochemistry (IHC). CXCL1 expressed in both the epithelial and stromal components of the HCC tissues. In the intratumor stroma, CXCL1 was expression was diffuse. In tumor cells, CXCL1 expression was mainly localized to the cytoplasm (Figure 1B). No significant difference in CXCL1 expression was observed between cells located in the tumor center and the invasive front area (Supplementary Figure S3). Seventy-seven of the patient samples had a high immunoreactive score (IRS) of 4, 5, or 6 (see Materials and Methods). The relationships between the CXCL1 expression level and clinical characteristics of HCC patients are summarized in Table 1 and Supplementary Table S1. CXCL1 expression was associated with perioperative alpha-fetoprotein (AFP) levels in the serum (*P* = 0.024) and tumor differentiation (*P* = 0.001). A Kaplan-Meier survival analysis was used to evaluate the relationship between clinical characteristics and both the three-year survival rate and disease-free survival rate. In regards to the survival rate, CXCL1 expression was a risk factor (*P* = 0.050) along with high TNM classification stages (*P* < 0.001), multiple tumors (*P* = 0.047), macrovascular invasion (*P* = 0.001), microvascular invasion (*P* = 0.001), high BCLC stages (*P* < 0.001), AFP ≥ 400 ng/mL (*P* = 0.036), poor differentiation (*P* = 0.024), and a class B Child-Pugh classification (*P* < 0.001) (Table 2). In relation to the three year disease recurrence, high CXCL1 (*P* = 0.001), high BCLC stages (*P* < 0.001), poor differentiation (*P* = 0.003), AFP ≥ 400 ng/mL (*P* = 0.003), microvascular invasion (*P* = 0.024), macrovascular invasion (*P* = 0.001), and high TNM stages (*P* = 0.004) were found to be risk factors. In the Cox proportional hazards regression model, CXCL1 (*P* = 0.008, RR = 2.139) was an independent risk factor for the three-year DFS rate with BCLC stage (*P* = 0.002, RR = 4.286), TNM stages (*P* = 0.049, RR = 3.564; *P* = 0.047, RR = 2.497), AFP level (*P* = 0.023, RR = 1.860), but not for the three-year survival rate (Table 2 and Supplementary Table S2). As CXCL1 is associated with AFP levels in the serum and with tumor differentiation, samples were further divided into groups: (1) AFP ≥ 400 ng/mL or (2) AFP < 400 ng/ mL; (1) High and moderate differentiation or (2) low differentiation. High CXCL1 expression was predictive of increased HCC recurrence in AFP < 400 ng/mL and high and moderate differentiation subgroups (Figure 1D, 1E). In order to confirm the IHC results, fresh-frozen HCC specimens, and the corresponding perioperative serum from the sixty-four patients in this group, were divided into recurrence and no recurrence. These samples were tested for CXCL1 expression by immunoblot and ELISA. Patients with recurrence (33/64) had higher expression of CXCL1 than those without recurrence (31/64) in both HCC tissues (*P* = 0.023) and serum (*P* = 0.011) (Figure 1F, 1G, 1H).

**Table 2:** Clinicopathological factors for prognosis by Univariate and Cox-multivariate regression analysis

Clinical demographic		Univariate analysis		Cox-multivariate analysis		Univariate analysis		Cox-multivariate analysis	
		3-year OS rate	*P*	HR (95% CI)	*P*	3-years DFS rate	*P*	HR (95% CI)	*P*
AFP	≥ 400/< 400	58.0/77.3	0.036	2.159 (1.040–4.482)	0.039	60.0/22.2	0.003	1.860 (1.091–3.111)	0.023
Microvascular invasion	Yes/No	45.0/80.8	0.001	1.865 (0.761–4.574)	0.173	28.1/43.8	0.024	1.722 (0.877–3.381)	0.114
Macrovascular invasion	Yes/No	20.0/74.2	0.001	0.677 (0.113–4.070)	0.670	0.0/41.5	0.001	1.924 (0.642–5.760)	0.242
TNM stage	I/II/III	81.3/65.3/44.4	< 0.001	2.473 (0.410–14.928) 0.928 (0.273–3.154)	0.324 0.905	48.7/33.4/19.5	0.004	3.564 (1.008–12.597) 2.497 (1.011–6.164)	0.049 0.047
Differentiation	Poorly/Moderately and Well	58.3/75.6	0.024	0.784 (0.352–1.747)	0.551	18.5/46.4	0.003	0.848 (0.460–1.562)	0.596
Tumor number	Single/Multiple	75.9/58.6	0.047	0.447 (0.105–1.901)	0.276	42.8/30.2	0.152		
Child-Pugh classification	A/B	80.3/36.4	< 0.001	6.905 (2.583–18.458)	< 0.001	41.2/30.3	0.222		
BCLC classification	A/B–C	82.7/43.2	< 0.001	14.346 (2.985–68.954)	0.001	47.9/17.1	< 0.001	4.286 (1.714–10.719)	0.002
CXCL1 expression	Low/High	81.4/66.5	0.050	1.779 (0.779–4.063)	0.172	57.8/30.4	0.001	2.139 (1.221–3.746)	0.008

### Effects of CXCL1 on cell metastatic behaviors

**Figure 2:**
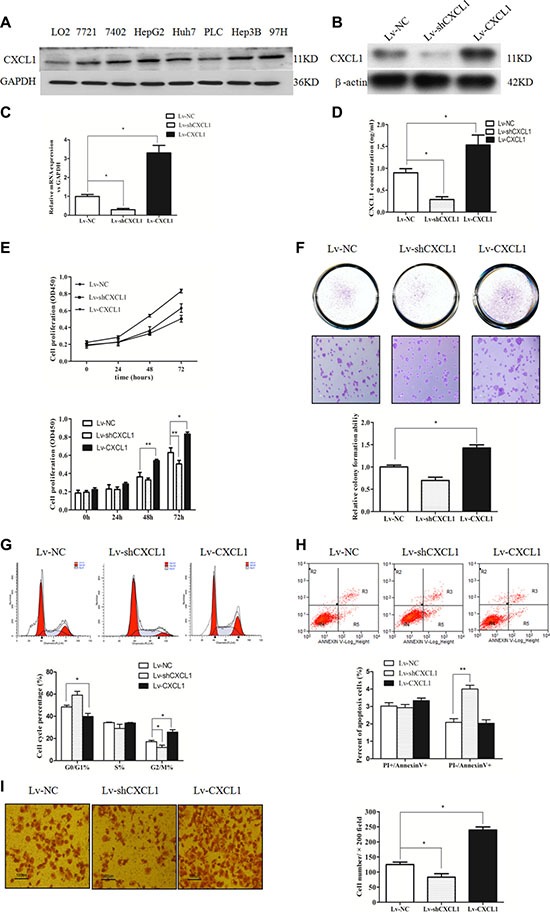
CXCL1 expression and the effect in hepatic cell and HCC cell lines (**A**) CXCL1 protein expression in hepatic cell and 7 HCC cell lines. (**B**, **C**, **D**) The effects of upregulating and downregulating CXCL1 in HepG2 cells. (**E**) Upregulating CXCL1 in HCC cells promoted proliferation, while downregulation inhibited proliferation. (**F**) Upregulating CXCL1 significantly increased the capacity for colony formation. In the downregulation group, no obvious differences were observed. (**G**) Upregulating CXCL1 led to G2/M stage arrest and downregulation significantly reduced cells in the G2/M stage. (**H**) Downregulation of CXCL1 induced more apoptosis in early stages. (**I**) Increasing CXCL1 expression promoted invasion into HepG2 cells, while decreasing CXCL1 expression inhibited invasion (original magnification × 200). Independent triplicate experiments were performed (mean ± SD). *P < 0.05, **P < 0.01.

CXCL1 expression was measured in one human normal liver cell line L02 and seven HCC cell lines. The expression of CXCL1 in all HCC cell lines was higher than in L02 (Figure 2A). The effect of modulating CXCL1 expression in HepG2 cells on the levels of protein and mRNA was measured (Figure 2B, 2C, 2D). Overexpression of CXCL1 promoted cell growth, inducing G2/M stage arrest and contributing to the invasion capacity, as determined by CCK-8, colony formation, cell cycle analysis, and matrigel invasion assays. CXCL1 knockdown inhibited HCC cell proliferation, reduced both the number of cells in the G2/M stage and number of cells that passed the matrigel membrane, and promoted apoptosis in early stages. There were no obvious differences observed in colony-forming capacity (Figure 2E–2I).

**Figure 3:**
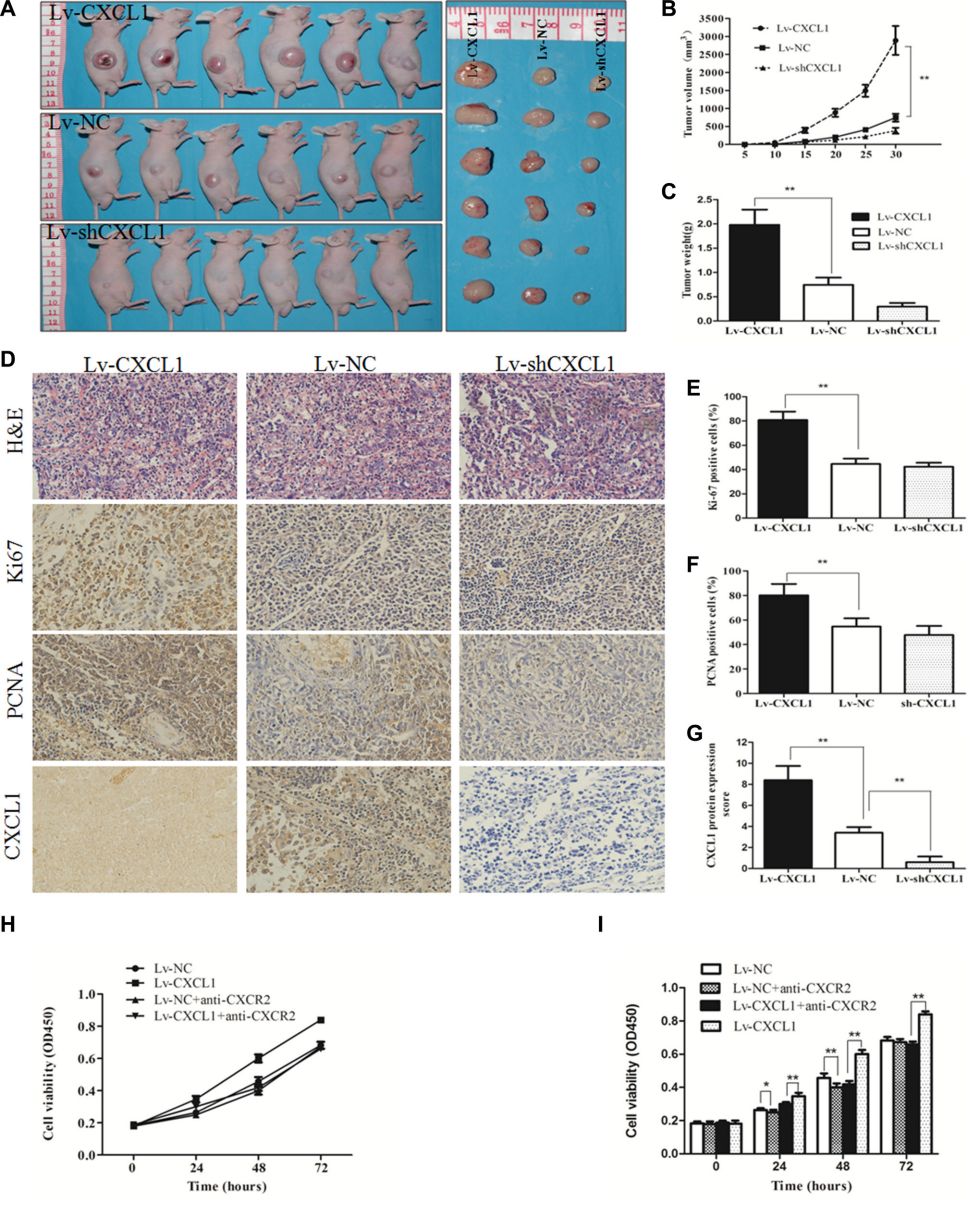
CXCL1 promoted tumorigenesis in a xenograft model (**A**) Images of tumors which were induced by the injection of Lv-CXCL1, Lv-shCXCL1 or Lv-NC cells into nude mice (*n* = 6). (**B**, **C**) Growth curve and histogram of tumor weights in nude mice. Increasing CXCL1 expression contributed to tumor growth in nude mice. (**D**) Images of tumors samples which were stained with hematoxylin and eosin. The expression of CXCL1, Ki-67, and PCNA were measured by IHC staining. (**E**, **F**, **G**): Upregulation of CXCL1 increased the expression of Ki-67 and PCNA. (**H**, **I**) The effect of CXCL1 on HepG2 cell growth via the autocrine pathway could be blocked by adding CXCR2 antibody at 5 μg/mL for 1 h. Triplicate experiments were performed independently (mean ± SD). *P < 0.05, **P < 0.01.

In order to verify the effect of CXCL1 on tumor progression, a xenograft tumor growth assay was performed by injecting nude mice with cells transfected with CXCL1 overexpression (Lv-CXCL1) or knockdown (Lv-shCXCL1) lentivirus or negative control (Lv-NC). Growth curves plotted from tumor volumes show that CXCL1 expression significantly promoted tumor growth (Figure 3A–3C). IHC staining of Ki-67 and proliferating cell nuclear antigen (PCNA) was performed on the tumors and, in comparison to the negative control, upregulation of CXCL1 significantly promoted proliferation, as indicated by the number of tumor cells positive for Ki-67 and PCNA staining. In contrast, downregulation of CXCL1 did not have obvious effects on growth compared to the control group (Figure 3D–3G). *In vitro* experiments revealed that CXCL1 promoted cell growth via autocrine pathways. Increasing CXCL1 expression promoted HepG2 cell growth, which was reversed by addition of the CXCL1-specific receptor antibody, CXCR2 (Figure 3H, 3I).

**Figure 4:**
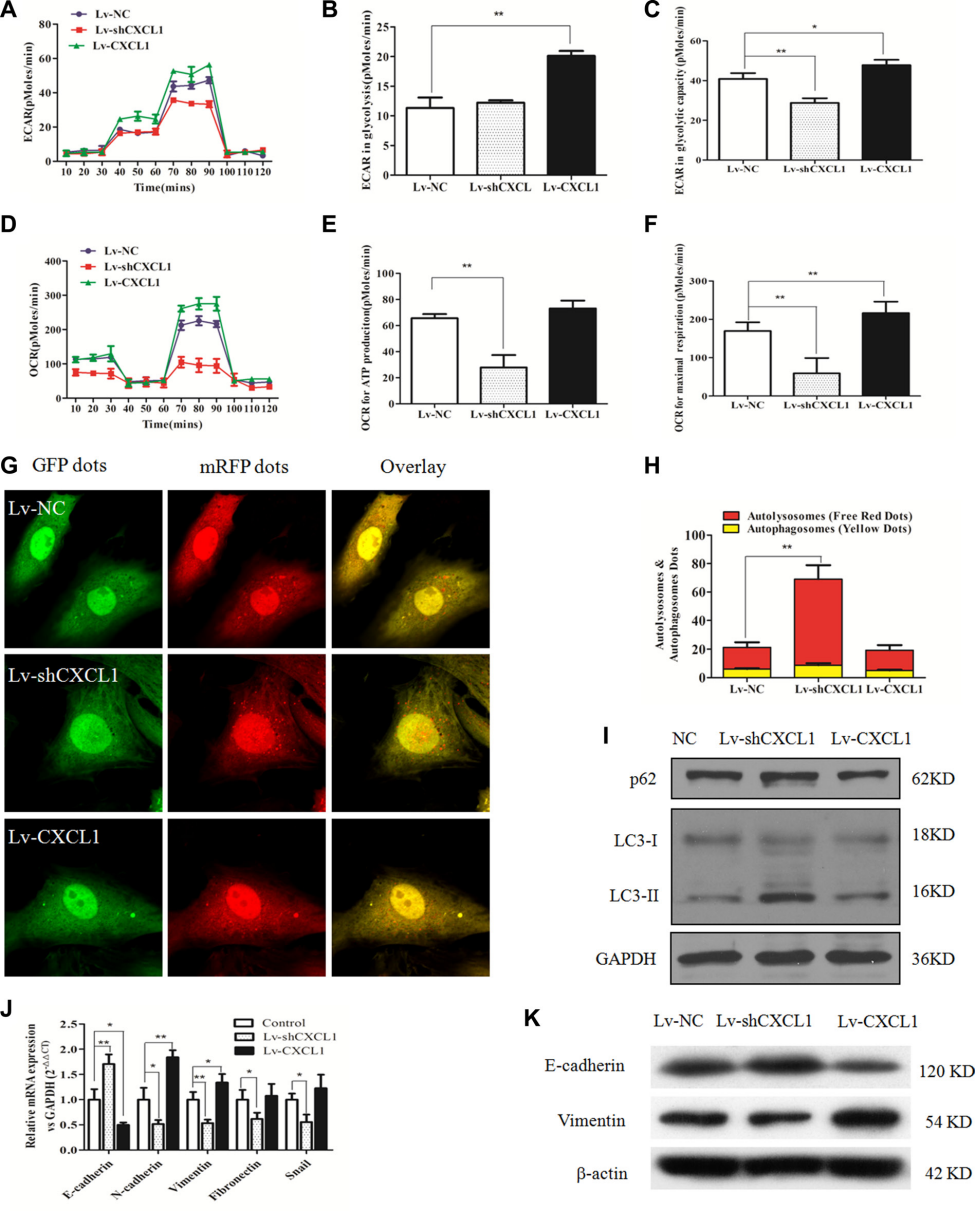
The effect of CXCL1 on mitochondrial respiration, autophagy and epithelial-mesenchymal transition (EMT) (**A**) ECAR curves in the Lv-CXCL1, Lv-shCXCL1, and NC groups. (**B**, **C**) Upregulating CXCL1 increased the level of glycolysis under normal conditions and the glycolytic capacity, while downregulating CXCL1 reduced the glycolytic capacity. (**D**) OCR curves in the Lv-CXCL1, Lv-shCXCL1, and NC groups. (**E**, **F**) CXCL1 enhanced the maximal respiration capacity, but not the level of ATP consumption under normal conditions, while inhibition of CXCL1 decreased both. (**G**, **H**) MRFP-GFP tandem fluorescent-tagged LC3 was employed to detect the effects of CXCL1 expression on autophagic flux under confocal microscopy. Yellow puncta represent autophagosomes, while red puncta represented autolysosomes. Decreasing CXCL1 expression induced more autophagic flux (**P < 0.01, one-way ANOVA) while no significant change was noted with increased CXCL1 expression. (**I**) Decreasing CXCL1 expression increased P62 levels and the ratio of LC3 II/LC3 I protein. (**J**, **K**) CXCL1 induced activation of the EMT pathway, while reducing CXCL1 suppressed this process as demonstrated by immunoblotting and qPCR. Three independent assays were performed (mean ± SD). *P < 0.05, **P < 0.01.

The oxygen consumption rate (OCR) and extracellular acidification rate (ECAR) in HCC cells indicated that reduced CXCL1 expression significantly suppressed OCR and oxidative phosphorylation (OXPHOS). In addition, glycolytic capacity was inhibited compared to the negative control, subsequently suppressing ATP and lactate production, which are essential for cell proliferation and survival. Increasing CXCL1 expression enhanced both of these capacities (Figure 4A–4F).

In order to demonstrate the effect of modulating CXCL1 expression on autophagy in HCC cells, a fluorescent mRFP-GFP-LC3 assay was used to assess LC3-I/II and P62 expression levels. Increasing CXCL1 expression did not induce obvious changes in red fluorescence, indicating that autophagy did not change in comparison to the Lv-NC group, while downregulation of CXCL1 greatly increased autophagy flux (Figure 4G, 4H). This conclusion was supported by the increased ratio of LC3-I/II in the knockdown cells (Figure 4I). In order to elucidate the function of CXCL1 in cell transformation, the effect of CXCL1 in activating the EMT pathway was evaluated. Upregulation of CXCL1 in HepG2 cells upregulated N-cadherin and vimentin, while suppressing E-cadherin, indicating stimulation of EMT. Conversely, suppressing CXCL1 expression inhibited EMT (Figure 4J, 4K).

### miR-200a targets CXCL1 expression and inhibits HCC cell growth

**Figure 5:**
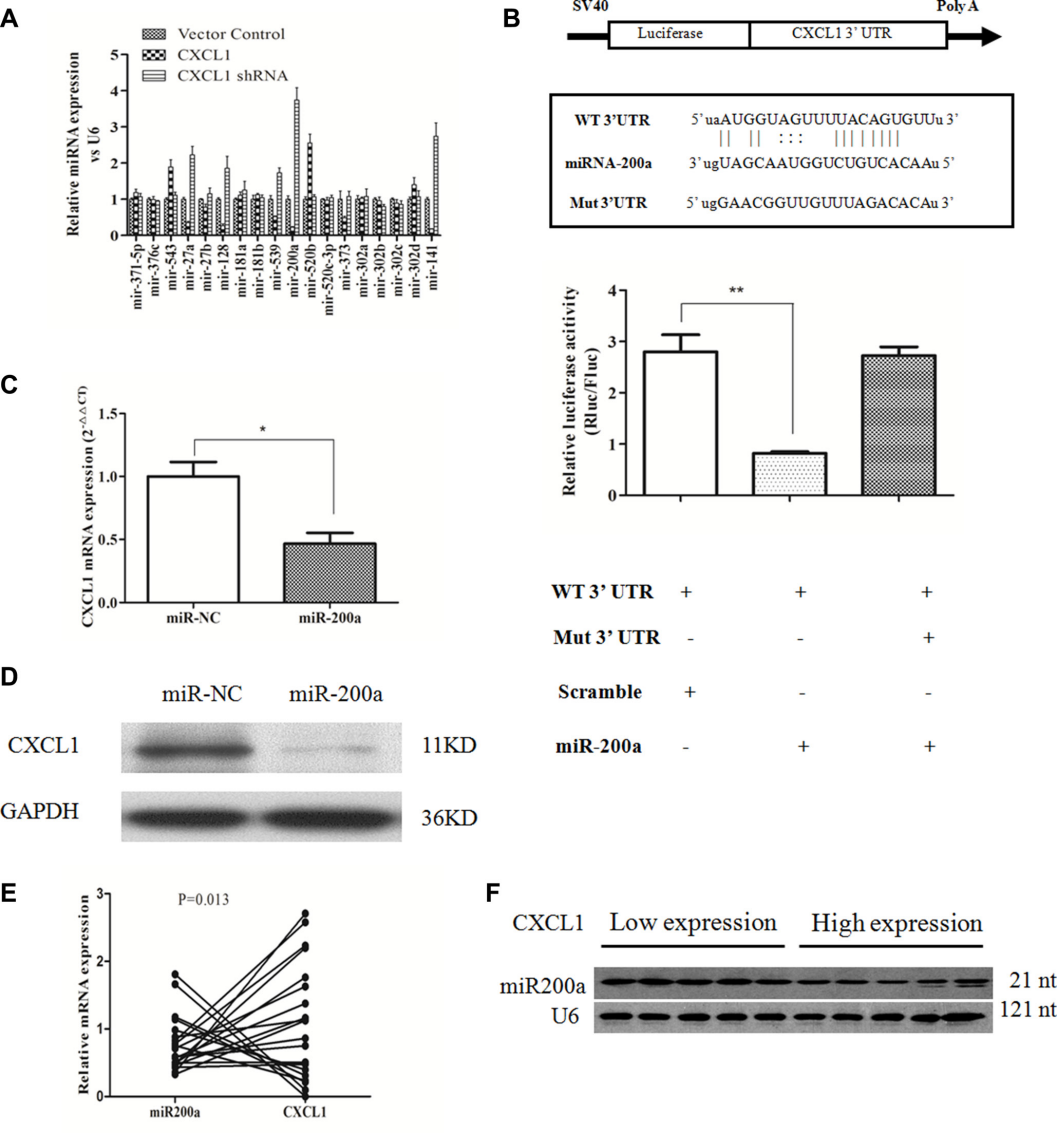
CXCL1 is a direct target of miR-200a (**A**) Candidate miRNAs that had a negative relationship with CXCL1 protein expression. (**B**) Possible binding sites for miR-200a in the CXCL1 mRNA and the effects of miR-200a and miR-NC in HepG2 cells, as demonstrated by luciferase reporter assay. (**C**, **D**) The effects of miR-200a on CXCL1 mRNA and protein expression. miR-200a decreased CXCL1 protein expression by downregulating the level of mRNA of CXCL1 in HepG2 cells. (**E**) The inverse relationship between the levels of CXCL1 mRNA and miR-200a in 20 frozen HCC specimens was analyzed by qPCR (*P* < 0.05, *r* = −0.543). (**F**) Representative image of the relationship between CXCL1 expression and miR-200a in frozen HCC tumor tissues as demonstrated by northern blot. Three independent assays were performed (mean ± SD). **P < 0.01,*P < 0.05.

miRNAs usually act as negative regulators of key biological functions in tumor progression by targeting certain types of gene expression [12]. Putative miRNAs that directly target CXCL1 mRNA were identified by performing an online search of the miRNA database (http://www.microrna.org/microrna/home.do) and TargetScan [13]. Of these candidate miRNAs, in addition to the having the capacity to bind the 3′ UTR of CXCL1 mRNA, miR-200a's expression was inversely proportional to the level of CXCL1 expression in HCC cells (Figure 5A). A luciferase reporter assay was conducted to verify this predicted interaction. Negative control (miR-NC) or a miR-200a mimic was co-transfected into HepG2 cells in combination with a luciferase reporter plasmid with the 3′-UTR of CXCL1 mRNA (containing the targeting miR-200a binding sites). miR-200a significantly reduced luciferase activity in comparison to miR-NC (Figure 5B). Furthermore, the expression of CXCL1 mRNA and protein were significantly reduced in the miR-200a mimic group in contrast to the miR-NC group (Figure 5C, 5D). This suggests that miR-200a partially inhibits CXCL1 expression by directly reducing the amount of CXCL1 mRNA. An inverse correlation between CXCL1 and miR- 200a was observed in frozen tumor tissues from 20 HCC patients by qPCR and northern blot (Figure 5E, 5F).

**Figure 6:**
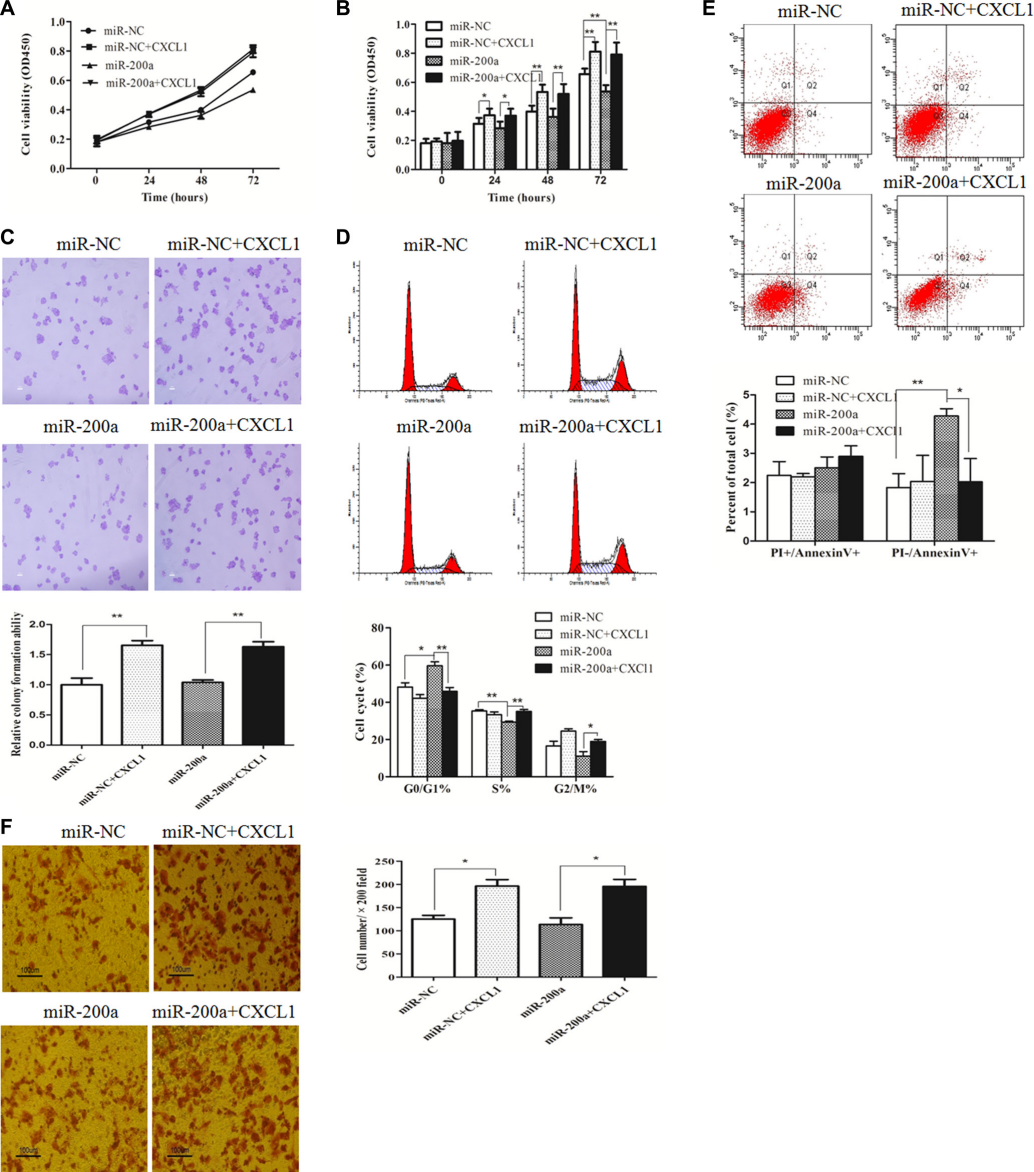
miR-200a inhibited HCC cell growth (**A**, **B**) The growth of HepG2 cells was significantly inhibited after transfection with miR-200a in comparison to the negative control, and this was reversed by adding exogenous CXCL1 protein. (**C**) Upregulating miR-200a expression induced G0/G1 stage arrest and reduced the percentage of cells in S stage. (**D**) Increasing numbers of apoptotic cells apoptosis in early stages were induced by inserting the miR-200a mimic in HepG2 cells, was rescued by adding exogenous CXCL1 protein. (**E**) Overexpression of miR-200a did not have remarkable effects on colony numbers, based on the colony formation assay. (**F**) No significant differences were observed in the invasion assay when miR-200a was upregulated in HepG2 cells. Triplicate experiments were performed independently (mean ± SD). *P < 0.05, **P < 0.01.

The function of miR-200a in HepG2 cells was assessed via proliferation, colony, apoptosis, and migration assays. The proliferation capacity of cells transfected with miR-200a mimic was significantly decreased in contrast to the negative control (Figure 6A, 6B). Transfection with miR-200a mimic increased the number of cells in early stage apoptosis and induced G0/G1 stage arrest, effectively inhibiting cells in S stage, which was similar to the observed results from suppressing CXCL1 expression (Figure 6D, 6E). Overexpression of miR-200a had no significant effects on colony-forming or invasion assays (Figure 6C, 6F). The biological effects induced by transfecting miR-200a plasmid into HepG2 cells could be reversed by addition of exogenous CXCL1(Figure 6).

## DISCUSSION

Chemokines secreted by inflammatory cells and tumor cells could not only recruit other inflammatory cells into the tumor microenviroment, but may also affect tumor growth, invasion, angiogenesis, and metastasis through corresponding cell surface receptors [14]. For example, CXCL1 contributes to the progression of various tumors by specifically binding to CXCR2 [15].

Many biomarkers, such as AFP in serum, tumor differentiation, BCLC classification, and MVI, have previously been identified as independent risk factors in HCC progression [16]. In this study, remarkable differences were observed in the levels of CXCL1 expression among tumor tissues by IHC staining. Level of CXCL1 protein in tumor, which was associated with AFP levels in serum and tumor differentiation, was an independent risk factor for recurrence along with AFP, BCLC classification, and TNM stages. This prognostic value was confirmed by protein level analysis in frozen tumor tissues and serum. In patients with serum AFP < 400 ng/mL or with moderately and highly differentiated tumors, high CXCL1 expression in tumors was correlated with higher rates of recurrence. However, no differences were found in CXCL1 expression levels between adjacent non-cancerous tissues and tumor tissues, in agreement with the data collected by Roessler [11]. This effect may be confounded by patients in this study that were also infected with viral hepatitis, where the tumor and non-tumor liver cells were located in the same liver inflammation background. These findings suggest that CXCL1 could be a predictive biomarker for recurrence in HCC patients.

Based on *in vitro* assay, CXCL1 promotes HCC cell proliferation, growth, and invasion; induces cell cycle arrest at G2/M stage; and decreases cell apoptosis. The oncogenic function of CXCL1 in HCC was confirmed by xenograft tumor assays in nude mice. We found that CXCL1 regulates mitochondrial metabolism, autophagy, and EMT pathways, all of which play crucial roles in tumor proliferation and invasion. Furthermore, miR-200a was identified as a negative regulator that modulates CXCL1 expression in HCC cells and tissues. Upregulation of CXCL1 significantly enhanced glycolysis and OXPHOS capacity in HCC cells. This increase in respiratory activity provides sufficient adenosine triphosphate and materials for tumor cell growth, while promoting tumor progression by reducing the pH of the tumor microenvironment through the end products of aerobic glycolysis [17–19]. Conversely, downregulation of CXCL1 expression caused inhibition of these capacities. The change in the mitochondrial metabolism pattern is a pivotal method to trigger autophagy and the EMT pathway [20, 21]. Downregulation of CXCL1 expression in HCC cells significantly increased the amount of autophagy flux. This enhancement in autophagy, in which intracellular organelles and proteins are recycled to drive ATP production and maintain homeostasis in tumor cells for survival in response to cell stress [22], may be partly due to energy deficiencies that are caused by suppressing mitochondrial metabolism. During tumor progression, glycolytic capacity has been revealed to be closely correlated with activation of EMT in tumor cells [21]. In this study, CXCL1 significantly activated the EMT pathway by upregulating the expression of E-cadherin, N-cadherin, and Vimentin in HCC cells and enhanced tumor cell invasion capacity, while reducing CXCL1 expression inhibited these processes. These results indicate that CXCL1 could be a potential target for treating HCC.

The development of HCC is caused by a complicated network of aberrant gene expression and microenvironment alterations, to which microRNAs may contribute [23]. Through bioinformatics methods, several miRNAs with the potential to target CXCL1 were identified. By comparing the expression levels of miRNAs with CXCL1 in specific cell types, miRNA -200a was selected as a promising effector; it was later determined to directly target the 3′UTR domain and decrease CXCL1 mRNA levels. In addition, miR-200a suppressed HCC cell proliferation and migration and induced apoptosis at early stages by decreasing CXCL1 protein levels post-transcriptionally. These results were further confirmed by rescue assays, in which adding exogenous CXCL1 protein overcame miR-200a inhibition. In tumor tissues, miR-200a levels were inversely proportional to CXCL1 mRNA. This indicates that miR-200a suppresses CXCL1 expression partly through enhancement of mRNA cleavage. For the HCC patients with high CXCL1 expression, who are at high risk for recurrence, miR-200a expression was usually low. Although miR-200a could potentially target other genes in HCC, these results are in agreement with previous observations [24–26] and indicate that miR-200a is a negative regulator of HCC progression and a potential biomarker for favorable prognosis.

In conclusion, these findings reveal that CXCL1 has an oncogenic role in HCC progression through activation of mitochondrial metabolism and EMT pathways. Meanwhile, miR-200a plays an anti-tumor role in HCC progression, in part by repressing CXCL1 expression. These results may provide potential new therapies for HCC targeting the miR-200a/CXCL1 pathway.

## MATERIALS AND METHODS

### Patients, specimens and cell lines

This study enrolled 119 primary HCC patients who had undergone hepatectomy between 2007 and 2012 and their diagnoses were confirmed by pathology methods. HBV infection was assessed by serum HBV surface antigen, hepatitis Be antigen, HBV core antibody, or HBV-DNA. Patients with long histories of alcohol intake, co-infection with hepatitis C virus, history of anti-tumor therapy prior to the hepatectomy, or extrahepatic metastasis confirmed by CT, MRI, or PET were excluded. The number of tumor nodules and the nodule diameters were confirmed by perioperative CT, MRI, or intraoperative ultrasound and postoperative measurements. R0 hepatectomy was performed on these patients, which was defined by tumor nodules that were completely resected, the distance from the sections to the margin of the tumors was ≥ 1 cm under direct observation, and clear histological margins were confirmed by pathology sections. Differentiation was characterized by the Edmondson grading system. Microvascular and macrovascular invasion were defined as in previous studies [18, 27]. All patients enrolled in this research gave their written consent in advance. The Ethics Committee of the Peking University People's Hospital assessed and approved the study.

Postoperative outpatient follow-ups were performed every 3 months at Peking University People's Hospital, including serum AFP, liver function, HBV-DNA level, chest X-ray, ultrasonography, or MRI. If the HBV DNA level was ≥ 1.00 × 10^3^ copies /mL, antiviral therapy was administered. The endpoint of this study was April 30, 2015. Survival time was measured from the date of first operation to the death date of the patient or the last follow-up. DFS time was the period between the operation day and the date when recurrence was been confirmed or the last follow-up date. If recurrence was diagnosed, a second hepatectomy, radiofrequency ablation, and transcatheter arterial chemoembolization were performed (Table 1).

The human HCC cell lines (HepG2, 7721, 7402, Huh7, PLC, Hep3B, and 97H) and the human hepatic cell line L02 were purchased from the Chinese Academy of Sciences (Shanghai Institutes for Biological Sciences, Shanghai, China). All cells were cultured in RPMI 1640 (Hyclone, Logan, UT, USA) supplemented with 10% fetal bovine serum (GIBICO, CA, USA) in 5% CO_2_ at 37°C. Sixty-four frozen-free tumor tissues and paired preoperative serum were collected from the HCC patients described previously.

### RNA expression measurement

Thirty paired frozen tumors and adjacent non-tumor tissues were randomly selected from the HCC patients described previously. The cells from Lv-NC, Lv-shCXCL1, Lv-CXCL1 cell groups were collected. In accordance with the manufacturer's instructions, RNA was isolated from these tissues and cells and used for quantitative RT-PCR. The list of primer sequences used in this research is included in Supplementary Table S3.

### Immunohistochemistry

Three μm thick sections were cut from the paraffin-embedded blocks. Sections were then deparaffinized and rehydrated. Staining was performed as previously described [28]. Prediluted polyclonal rabbit anti-CXCL1 antibody (1:1500, Abcam Co., Hong Kong, China), polyclonal rabbit anti-CXCL1 antibody (1:2500, Abcam Co., Cambridge, MA, USA), polyclonal rabbit PCNA antibody (1:100, Abcam Co., Cambridge, UK), monoclonal rabbit anti-Ki-67 antibody (1:800, Abcam Co., Cambridge, MA, USA), monoclonal mouse antibody (1:500, CST Co., Danvers, MA, USA) were added and incubated for 16 h at 4°C. The negative control was established at this step, adding PBS was instead of the antibody. Secondary antibody at working dilutions (rat anti-rabbit IgG, goat anti-mouse IgG, Beijing XiYa Jinqiao Biology Technology Company, Beijing, China) was added, followed by the color reaction. Immunohistochemical staining was evaluated by two independent pathologists that did not have access to the patients' clinical information (L.H. Qian and J.Q. Song) in Peking University People's Hospital. Briefly, the location of immunoreactivity (cytoplasm, cell membrane) was noted. In order to evaluate CXCL1 expression in tumor cells, the expression level in each section was scored based on the intensity and density of immunoreactive cells, which was defined by the immunoreactive score (IRS), the sum of the density and intensity of stained cells. The staining intensity was scored where negative = 0, weak = 1, moderate = 2 and strong = 3. The percentage of stained cells was semi-quantitatively estimated as 0 ≤ 1% of cells, 1 = 1–40%, 2 = 40–75%, 3 ≥ 75%. The IRS ranged from 0 to 6. The CXCL1 protein expression levels were divided into two groups based on the collected scores. Low expression was score < 4, while high expression was score ≥ 4 [9] if there were any discrepancies between the scores. The average scores from each were recorded.

### Western blot and ELISA

Extraction of total protein from HCC tissues and cell lines, as well as immunoblotting assays, was performed as previously described [29]. Nitrocellulose membranes were incubated with rabbit polyclonal anti-CXCL1 antibody (1:1200, Abcam, Cambridge, MA, USA), rabbit polyclonal β-actin antibody (1:1500, Abcam, Cambridge, MA, USA), rabbit monoclonal anti- GAPDH (1:2000, Cwbiotech, Beijing, China), monoclonal mouse anti-P62 antibody (1:2000, Abcam Co., Cambridge, MA, USA), rabbit polyclonal anti-LC3II (1:1000, Abcam Co., Cambridge, MA, USA), mouse monoclonal anti-E-cadherin (1:1000, CST, Danvers, MA,USA), mouse monoclonal anti-vimentin (1:3000, Abcam Co., Cambridge, MA, USA). Horseradish peroxidase-conjugated anti-rabbit IgG antibody or -conjugated goat anti-mouse IgG were added as the secondary antibody (1:4000; Zhongshan Jingqiao Biology Technology Co., Beijing, China). For ELISA (enzyme linked immunosorbent assay), serum was collected from the patients preoperatively. CXCL1 expression levels were measured in duplicate using an ELISA kit (R&D Systems, Wiesbanden, Germany) according to the manufacturer's instruction.

### Genetic manipulation

For manipulating the expression of CXCL1 in the HepG2 cell line, pools of concentrated recombined lentivirus vectors, pLVX-shRNA1, pLVX-IRES-PURO (Clontech Laboratories Inc., CA, USA) with targeting genes were constructed according to the protocols, and an empty shRNA vector was used as a negative control (sequences are listed in Supplementary Table S3). Tumor cells were plated in six-well plates at a concentration of 5 × 10^5^ cells/well, and were then transfected with lentivirus (MOI = 10). After 48 h, 2 μg/mL puromycin (Sigma-Aldrich, St. Louis, MO, USA) was added to the cells. Once the cells reached 70% confluence, they were passaged and then cultured with puromycin for 2 weeks to select stable cell lines. These stable cells were designated as lentivirus vector (Lv)-NC cells (negative control group), Lv-CXCL-shRNA (downregulation group) and Lv-CXCL1 (upregulation group). The oligonucleotide of miR-200a (5′-GGGACCCCACGT CCCTCCCGGGCCCCTGTGAGCATCTTACCGGACA GTGCTGGATTTCCCAGCTTGACTCTAACACTGTCT GGTAACGATGTTCAAAGGTGACCCGCCGCT-3′) and negative control (miR-NC) (5′-UUGUACUACACAAAA GUACUG-3′) were constructed, amplified, and transfected into HepG2 cells as previously described [30].

### Cell proliferation assay

Cell Counting Kit 8 (CCK8) and colony formation assays were employed to evaluate cell proliferation. In the CCK8 assays, cells were plated into 96-well plates at a density of 2 × 10^3^ cells/well, and cultured at 37°C for 24 h. CCK8 was added to the wells and incubated for 1.5 h. The optical density in each well was measured by a Biotek Elx800 microplate reader (Bio-tek, Currumbin VT, USA) at 450 nm. In the colony formation assay, different groups of cells were plated at a density of 1 × 10^3^ cells/well into the 6-well plates (containing 0.6% base agar and 0.3% top agar) and incubated at 37°C for 15 days and the cells were finally stained with 0.5% crystal violet (Sigma-Aldrich, St. Louis, MO, USA). CXCL1 protein (Abcam Co., Cambridge, MA, USA) was added to specific wells at 100 ng/mL for 2–4 h. Chemokine (C-X-C motif) receptor 2 (CXCR2) antibody (Abcam Co., Cambridge, MA, USA) was added to antagonize the effects of CXCL1 at 5 μg/mL for 1 h [31].

### Cell cycle assay

Once cells had grown to 80% confluence, they were washed with ice-cold PBS and fixed in 70% ethanol overnight at 0°C. Cells were resuspended with 25 μL of 2 mg/mL propidium iodide, 10 μL of 10 mg/mL RNaseA, and 1,000 mL of PBS and kept at 4°C in the dark for 1 h. The cells were then separated and assessed using a FACSCalibur (BD, Franklin Lake, NJ, USA). Experiments were performed in triplicate for each group.

### Apoptosis assay

Cells were harvested at ∼80% confluence and washed with ice-cold PBS solution. The cells were resuspended in 400 μL of 1× binding buffer, 1 mL of 1× staining buffer and 5 μL of Annexin V-APC (Ebioscience, San Diego, CA, USA) and incubated for 15 min in dark at 4°C. Cells were analyzed using a FACSCalibur (BD). Experiments were conducted in triplicate for each group.

### Matrigel invasion assay

Assays were performed with a Chemicon Cell Invasion Assay Kit ECM550 (Chemicon, Temecula, CA, USA) as described by the manufacturer. Cells (2.5 × 10^4^ cells/well) were resuspended with 200 μL of RIPM 1640 without FBS and cultured in the upper chamber. In the bottom chamber, 500 μL of RIPM 1640 with 10% FBS was added. The cells were incubated for 48 h at 37°C, followed by removal of cells from the upper surface. Membranes were immersed in 4% paraformaldehyde, stained with 0.5% crystal violet and mounted. This assay was repeated in triplicate. Photography was performed using an Olympus inverted microscope.

### Tumorigenicity assay

Three groups of HepG2 cells (approximately 10^7^ cells in 200 μL of RPMI-1640 serum-free medium) were injected into the right renal capsule of each 4–8 week old female BALB/C nude mouse (Vital River Laboratory Animal Technology Co., Beijing, China). Each group included six mice. Tumor diameters were measured every 3 d to evaluate tumor growth. Both the length and width of the tumors were measured with a slide caliper. Tumor volume was calculated using the formula V = 1/2 (L × W^2^). Mice were sacrificed 30 d after injection, and tumors were collected and weighed. Animals were maintained in a pathogen-free environment. All animal studies and protocols were approved by the Peking University People's Hospital Animal Care committee according to Peking University People's Hospital animal use guidelines.

### Metabolism analysis

Cellular respiration function was evaluated using an XF-24 analyzer (Seahorse Biosciences Inc., MA, US). The assays were performed according to the XF cell mito stress test kit instructions (Seahorse Bioscience Inc., MA, US). Cells were plated at a density of 1.5 × 10^4^ cells/well and incubated overnight at 37°C, followed by XF bioenergetic assay. The mitochondrial respiration test was conducted by adding reagents as follows: 1 μM oligomycin, 1 μM carbonyl cyanide-p-trifluoromethoxyphenylhydrazone, 1 μM rotenone, and 1 μM antimycin A according to the manufacturer's instruction. The OCR and extracellular acidification rate ECAR were measured in triplicate.

### Autophagic flux analysis

Cells were transfected with mRFP-GFP-LC3 plasmid (HanBio, Shanghai, China)) at a density of 1 × 10^4^ cells/ well according to the manufacturer's instructions. After culturing for 24 hours, cells were fixed with 4% paraformaldehyde and cells were detected under a confocal microscope (Leica TCS SP8 MP FLIM, Leica, Wetzlar, Germany) with proper fluorescence filter sets. Autophagic flux was measured by quantifying the average number of yellow (autophagosomes) and red puncta (autolysosomes) in three different cells for each group [32]. The experiment was performed in triplicate.

### Northern blot

Formaldehyde denaturing agarose gel electrophoresis was employed to analyze the total amounts of RNA [33]. After the RNA was transferred to a Hybond N^+^ nylon membrane (Amersham, Freiburg, Germany), ultraviolet light was used for detection. miR- 200a antisense, 5′-ACATCGTTACCAGACAGAGTTA-3′ (Sunny Biotechnology Co., Ltd, Shanghai, China) labeled with the Prime-a-Gene Labeling System (Promega, Madison, WI, USA), was used to hybridize with miR-200a for 16 hrs after prehybridization for 3 hrs at 42°C. The membranes were then exposed to a Kodak XAR-5 film for 48 hrs. Human U6 snRNA was used as the positive control. The probe sequence was 5′-GCAGGGGCCATGCTAATCTTCTCTGTATCG-3′.

### Luciferase reporter assay

The human CXCL1 3′-UTR region, which contains the putative binding sites for miR200a, was amplified by PCR and inserted into the psiCHECK-2 vector (Promega, Madison, WI, USA) between the restrictive sites Xho I and Not I, and the insertion was validated by sequencing. A mutation of the miR-200a binding site was also generated using mutagenic oligonucleotide primers. The dual luciferase assays were used to confirm direct binding and function between miR-200a and the CXCL1 3′-UTR region (wild or mutant type), and the assays were performed according to the manual (Promega, Madison, WI, USA) [34].

### Statistical analysis

SPSS 17.0 software (SPSS. Inc., IL, USA) and Graph Pad Prism 5.0 (GraphPad Software. Inc., CA, USA) software were employed for statistical analysis and plotting data graphically. Continuous parameters were presented as mean ± SD. Groups of three were analyzed by one-way ANOVA. Student's test was performed to analyze data in groups of two in the cell experiments. A non-parameter test was used to determine associations among clinicopathologic variables. Differences between qualitative variables were compared with the Chi-square test (Pearson test) or Fisher exact test. Survival curves were plotted by the Kaplan-Meier method. A log-rank test was used to compare the differences. Multivariate analyses were conducted with the Cox proportional hazards regression model. Statistically significant difference was set at *P* < 0.05. A comparison of the mRNA expression levels of CXCL1 in a cohort of 247 mostly HBV-associated HCC patients (236/247) was conducted between tumor and adjacent non-tumor tissues. These data came from the microarray database at www.Oncomine.org, which was established by Roessler et al. on the Affymetrix Human Genome HT U133A Array [11].

## SUPPLEMENTARY MATERIALS FIGURES AND TABLES


