# Supplementary material for: Elevated CXCL1 increases hepatocellular carcinoma aggressiveness and is inhibited by miRNA-200a
Source: Oncotarget. 2016 Aug 17;7(40):65052–66. doi: 10.18632/oncotarget.11350 (PMC5323138; doi:10.18632/oncotarget.11350)
Supplement: Supplementary file 1 [file oncotarget-07-65052-s001.pdf]

Elevated CXCL1 increases hepatocellular carcinoma aggressiveness and is inhibited by miRNA-200a

Supplementary Materials

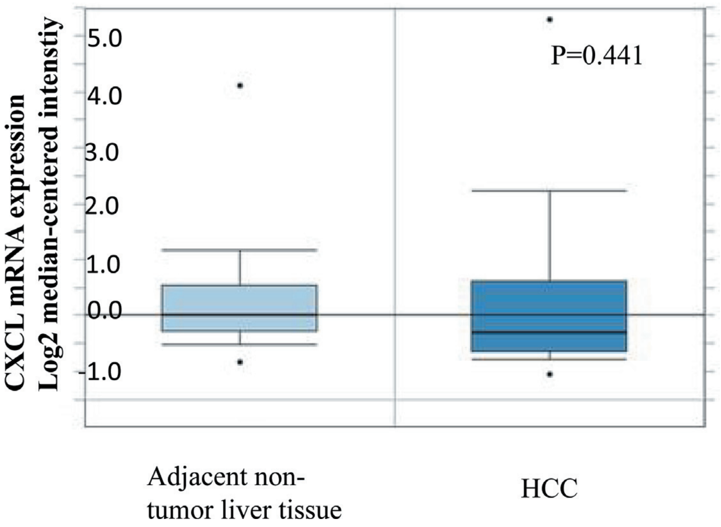

Supplementary Figure S1: The comparison of CXCL1 mRNA expression between HCC and adjacent non-tumor tissues from 247 HCC patients, were obtained from the microarray database (www.Oncomine.org) which was conducted by Rossler. Reply: This graph was cited from the public dataset (www.Oncomine.org), which is free to the public. But if we want to export the original graph of this data, subscription edition of Oncomine is required. In our institution, we do not subscribed this edtion.

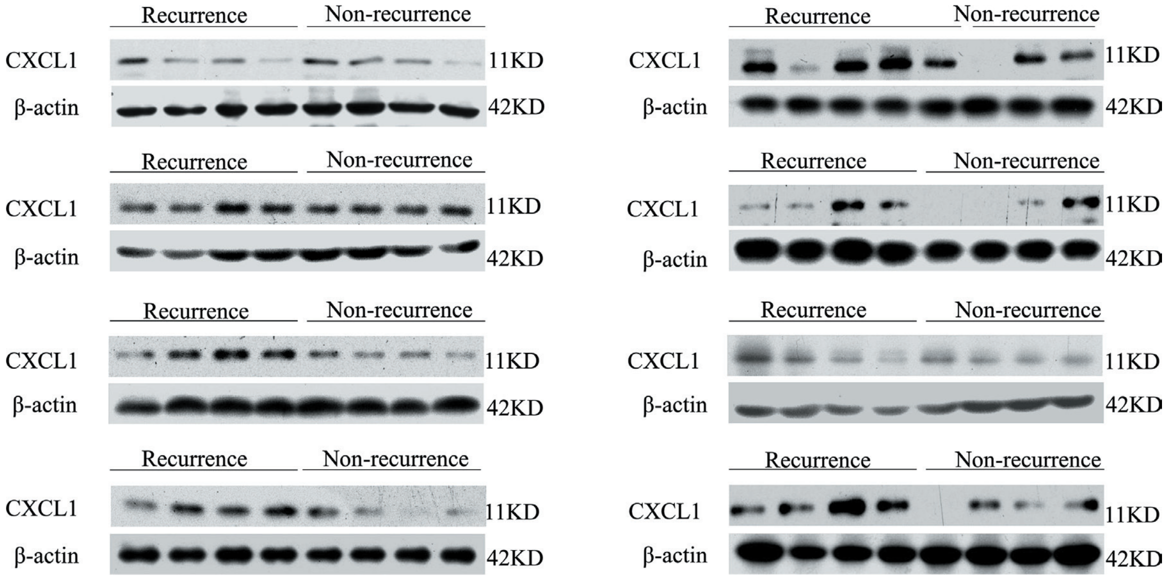

Supplementary Figure S2: CXCL1 expression in patients with or without recurrence (33 vs 31) in 3 years after hepatecotmy, which was tested by western blot.

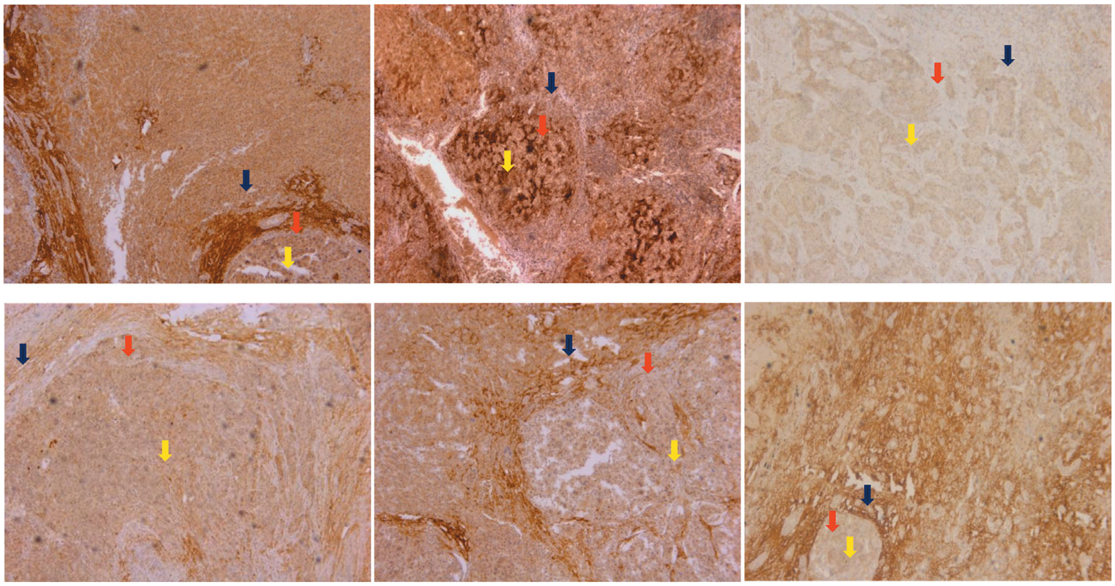

Supplementary Figure S3: CXCL1 protein expression in different areas of the tumor. Yellow arrow: center of the tumor, red arrow: the invasive front of the tumor, blue arrow: the stroma tissues around the tumor. Original magnification was 50×.

Supplementary Table S1: Associations between CXCL1 expression and clinicopathologic variables of HCC patients *n* (%)

Clinical characters	CXCL1			*P*-value
	All case *n* = 119	High expression	Low expression	
Gender(Female/Male)				
Female	19	13 (16.9)	6 (14.3)	0.712
Male	100	64 (83.1)	36 (85.7)	
Age (≥ 60/< 60)				
≥ 60	69	49 (63.6)	20 (47.6)	0.091
< 60	50	28 (36.4)	22 (52.4)	
Cirrhosis				
No	18	9 (11.7)	9 (21.4)	0.156
Yes	101	68 (88.3)	33 (78.6)	
AFP (≥ 400/< 400)				
≥ 400 ng/mL	35	28 (36.4)	7 (16.7)	0.024
< 400 ng/mL	84	49 (63.6)	35 (83.3)	
HBV-DNA (Positive/Negative)				
Positive	54	35 (45.5)	19 (45.2)	0.982
Negative	65	42 (54.5)	23 (54.8)	
Microvascular invasion (Yes/No)				
Yes	32	23 (29.9)	9 (21.4)	0.321
No	87	54 (70.1)	33 (78.6)	
Macrovascular invasion (Yes/No)				
Yes	5	5 (6.5)	0 (0.0)	0.160*
No	114	72 (93.5)	42 (100.0)	
TNM stage (I/II/III)				
I	67	38 (49.4)	29 (69.0)	0.099
II	28	20 (26.0)	8 (19.0)	
III	24	19 (24.7)	5 (11.9)	
Differentiation (Poorly/Moderately-Well)				
Poorly	27	25 (32.5)	2 (4.8)	0.001
Moderately-Well	92	52 (67.5)	40 (95.2)	
Tumor number (Single/Multiple)				
Single	90	56 (72.7)	34 (81.0)	0.318
Multiple	29	21 (27.3)	8 (19.0)	
Encapsulation (Invasion/Non-invasion)				
Invasion	43	5 (6.5)	38 (90.5)	0.719*
Non-invasion	76	72 (93.5)	4 (9.5)	
Tumor size (≥ 5 cm/< 5 cm)				
≥ 5 cm	54	36 (46.8)	18 (42.9)	0.683
< 5 cm	65	41 (53.2)	24 (57.1)	
Child-Pugh classification (A/B)				
A	108	69 (68.9)	39 (92.9)	0.745*
B	11	8 (10.4)	3 (7.1)	
BCLC classification (A/B–C)				
A	86	53 (68.8)	33 (78.6)	0.257
B–C	33	24 (31.2)	9 (21.4)	

*Fisher exact test.

Supplementary Table S2: Clinicopathological factors for prognosis by Univariate and Cox-multivariate regression analysis

Clinical demorgraphic		Univariate analysis		Cox-multivariate analysis		Univariate analysis		Cox-multivariate analysis	
		3-year OS rate	*P*	HR (95% CI)	*P*	3-years DFS rate	*P*	HR (95% CI)	*P*
Gender	Femal/Male	56.8/74.6	0.093			30.7/41.6	0.085		
Age	≥ 60/< 60	69.6/74.5	0.429			47.1/53.7	0.262		
Cirrhosis	Yes/No	77.0/64.2	0.474			42.2/28.4	0.076		
AFP	≥ 400/< 400	58.0/77.3	0.036	2.159 (1.040–4.482)	0.039	60.0/22.2	0.003	1.860 (1.091–3.111)	0.023
HBV-DNA	Positive/Negative	72.0/71.3	0.960			42.8/37.4	0.492		
Microvascular invasion	Yes/No	45.0/80.8	0.001	1.865 (0.761–4.574)	0.173	28.1/43.8	0.024	1.722 (0.877–3.381)	0.114
Macrovascular invasion	Yes/No	20.0/74.2	0.001	0.677 (0.113–4.070)	0.670	0.0/41.5	0.001	1.924 (0.642–5.760)	0.242
TNM stage	I/II/III	81.3/65.3/44.4	< 0.001	2.473 (0.410–14.928)<br>0.928 (0.273–3.154)	0.324<br>0.905	48.7/33.4/19.5	0.004	3.564 (1.008–12.597)<br>2.497 (1.011–6.164)	0.049<br>0.047
Differentiation	Poorly/ Moderately and Well	58.3/75.6	0.024	0.784 (0.352–1.747)	0.551	18.5/46.4	0.003	0.848 (0.460–1.562)	0.596
Tumor number	Single/Multiple	75.9/58.6	0.047	0.447 (0.105–1.901)	0.276	42.8/30.2	0.152		
Encapsulation	Invasion/ non-invasion	55.6/78.0	0.229			38.1/39.8	0.896		
Tumor size	≥ 5 cm/< 5 cm	69.1/73.8	0.308			27.3/48.4	0.367		
Child-Pugh classification	A/B	80.3/36.4	< 0.001	6.905 (2.583–18.458)	< 0.001	41.2/30.3	0.222		
BCLC classification	A/B - C	82.7/43.2	< 0.001	14.346 (2.985–68.954)	0.001	47.9/17.1	< 0.001	4.286 (1.714–10.719)	0.002
CXCL1 expression	Low/High	81.4/66.5	0.050	1.779 (0.779–4.063)	0.172	57.8/30.4	0.001	2.139 (1.221–3.746)	0.008

Supplementary Table S3: Oligonucleotide primers for genes were used in this research

Gene	oligonucleotide primers
CXCL1	F-5′TGCTGAACAGTGACAAATCCAAC3′<br>R-5′TGGGGTTGACATTTCAAAAAGAA3′
Small heparin RNA for CXCL1	Top strand 5′-GATCCGAATGGGCGGAAAGCTTGCCTCAATTTCAAGAGAA TTGAGGCAAGCTTTCCGCCCATTCTTTTTTG-3′,<br>Bottom strand 5′-AATTCAAAAAAGAATGGGCGGAAAGCTTGCCTCAATT CTCTTGAAATTGAGGCAAGCTTTCCGCCCATTCG-3′.
For Lv-CXCL1, insert DNA sequence	5′-ATGGCCCGCGCTGCTCTCTCCGCCGCCCCCA GCAATCCCCGGCTC CTGCGAGTGGCACTGC TGCTCCTGCTCCTGGTAGCCGCTGGCCGGCG CGCAGCAGGAGCGTCCGTGGCCACTGAAC TGCGCTGCCAGTGCTTG CAGACCCTGCAGG<br>GAATTCACCCCAAGAACATCCAAAGTGTGA ACGTGAAGTCCCCCGGA CCCCACTGCGCCC<br>AAACCGAAGTCATAGCCACACTCAAGAATG GGCGGAAAGCTTGCCTC AATCCTGCATCCCC<br>CATAGTTAAGAAAATCATCGAAAAGATGC<br>TGAACAGTGACAAATCCAACTGA-3′
microRNA-200a probe	5′-ACATCGTTACCAGACAGAGTTA-3′
U6 probe	5′-GCAGGGGCCATGCTAATCTTCTCTGTATCG-3′
MiR-200a	GSP:5′GGGTTTTAACACTGTCTGGTA3′<br>R:5′CAGTGCGTGTCGTGGA3′
GAPDH	F 5′-TGACTTCAACAGCGACACCCA-3′<br>R 5′-CACCCTGTTGCTGTAGCCAAA-3′
E-cadherin	F 5′-CTTTGACGCCGAGAGCTACA-3′<br>R 5′- TTTGAATCGGGTGTCGAGGG-3′
Vimentin	F 5′-GGACCAGCTAACCAACGACA-3′<br>R 5′-AAGGTCAAGACGTGCCAGAG-3′
N-cadherin	F 5′-CGCTATTTGTCATCAGCTCGC-3′<br>R 5′- TGCGATTTCACCAGAAGCCT-3′
Fibronectin	F 5′- ACAAGCATGTCTCTCTGCCA-3′<br>R 5′- TTTGCATCTTGGTTGGCTGC-3′
Snail	F 5′-GAGGACAGTGGGAAAGGCTC-3′<br>R 5′- TGGCTTCGGATGTGCATCTT-3′
